# Authors’ Response to Peer Reviews of “Evaluating the Financial Factors Influencing Maternal, Newborn, and Child Health in Africa: Tobit Regression and Data Envelopment Analysis”

**DOI:** 10.2196/85578

**Published:** 2025-11-28

**Authors:** Youssef Er-Rays, Meriem M'dioud, Hamid Ait-Lemqeddem, Badreddine El Moutaqi

**Affiliations:** 1 Polydisciplinary Faculty of Larache, Abdelmalek Essaadi University, Tetouan, Morocco, 212 5399-79316; 2 École nationale des sciences appliquées, Ibn Tofail University, Kenitra, Morocco

**Keywords:** financial determinants, maternal, newborn, and child health, health care efficiency, Africa, health expenditure, data envelopment analysis, Tobit regression

*This is the authors’ response to peer-review reports for “Evaluating the Financial Factors Influencing Maternal, Newborn, and Child Health in Africa: Tobit Regression and Data Envelopment Analysis.”*

## Round 1 Review

### Reviewer GP [1]

The manuscript [2] provides a valuable contribution to the understanding of health care system efficiency in Africa, particularly in the context of maternal, newborn, and child health (MNCH). The study is ethical, with appropriate use of data from reputable sources such as the World Health Organization, and the methods employed—data envelopment analysis and Tobit regression—are suitable for assessing the technical efficiency of health care systems across 46 African countries.

The material is original, and the paper addresses a significant gap in the literature by focusing on the financial and efficiency factors impacting MNCH in Africa. Related work is discussed and cited adequately, although a few more recent studies could be included to strengthen the literature review.

The writing is generally clear, though there are some areas where the discussion of the results could benefit from more detail. The study methods are appropriate for the research objectives, and the data used appear to be valid and reliable. The findings are significant and present actionable insights for policymakers, especially in terms of understanding the inefficiencies in the health care systems that impact MNCH outcomes.

The conclusions are reasonable and are supported by the data, although more detailed recommendations for practical application could enhance the paper’s impact. The topic is certainly of interest to the readership, as it addresses key issues surrounding health care efficiency and the achievement of Sustainable Development Goal 3 in Africa.

Overall, I recommend the manuscript for publication with minor revisions to improve the clarity of some sections and provide more detailed policy recommendations.

**Response:** The authors express gratitude for the constructive comments on the manuscript, recognizing the study’s contribution to understanding health care system efficiency in Africa, particularly in MNCH. The authors have clarified and expanded the discussion of the results, updated the literature review to include recent studies, and added more concrete policy recommendations in the Conclusion section. These changes aim to strengthen the theoretical framework and align the research with the existing body of work. The authors thank the reviewers for their feedback for improving the manuscript’s quality and clarity.

### Reviewer GW [3]

#### General Comments

Although the title might initially seem misleading, the paper tackles an essential issue of efficiency in delivering MNCH services in Africa. Given the well-known challenges facing maternal and newborn health in the region, the importance of this study cannot be overstated.

#### Specific Comments

##### Major Comments

1. The whole abstract is more about general efficiency in health care systems and less about the financial factors influencing MNCH in Africa. The title has to reflect what the study actually presents.

**Response:** Thank you for this comment. We will address it.

2. If the key aim of the study was to determine how financial factors influence MNCH, one would then expect to see, in the abstract, the extent of the influence of financial factors such as health expenditure, coverage index, and expenditure per capita.

**Response:** Thank you for this comment. We will address it.

3. The Introduction section starts with a presentation of the number of women dying in 2020 and the number of children dying in 2021. These data are not supported with any citations. It is also a little strange that for the number of women dying, the study refers to 2020 data, while for that of children, the study refers to 2021.

**Response:** We greatly appreciate your keen observation on the limitations of the data. You are absolutely right in your assessment. The primary challenge we encountered during this analysis was indeed the unavailability of comprehensive, directly reported numbers for maternal and newborn deaths specifically for the entire African continent in both 2020 and 2021.

- The Introduction section starts with a presentation of the number of women dying in 2020 and the number of children dying in 2021. These data are not supported with any citations. It is also a little strange that for the number of women dying, the study refers to 2020 data, while for that of children, the study refers to 2021.
- **Response:** We greatly appreciate your keen observation on the limitations of the data. You are absolutely right in your assessment. The primary challenge we encountered during this analysis was indeed the unavailability of comprehensive, directly reported numbers for maternal and newborn deaths specifically for the entire African continent in both 2020 and 2021.

4. The Introduction section does not provide sufficient motivation for investigating efficiency in health systems. The first paragraph presents the maternal health challenges, while the second paragraph quickly goes to the methods for establishing efficiency. There is no connection as to why investigating efficiency is necessary.

**Response:** Thank you for this comment. We will address it.

5. The Introduction section provides a descriptive review of other studies without depth. It lists the different studies without synthesizing them. It would be useful if they were at least lifted up to present issues/themes so it is easy to connect with what the study is about.

**Response:** Thank you for this comment. We will address it.

6. There is a sentence in the Methods section (Data Sources and Variables) that says “Input, output, and explanatory variables were selected to assess the accuracy of the WHO [World Health Organization]...” Are there three types of variables in your study?

**Response:** Thank you for this comment. Yes, we show them in Table 1.

7. What is presented as stages of data envelopment analysis does not go further to describe how the study made use of these stages. Much of the presentations are about what these stages are and sometimes the historical background. It would be useful to put more emphasis on how the study used these stages so it assures the credibility and reliability of the findings.

**Response:** Thank you for this comment. We will address it.

8. The presented results do not have a clear foundation from the methods. The chain of evidence from the data is lacking, from their processing to their results.

**Response:** Thank you for this comment. We will address it.

9. The parameters presenting the results are not clearly defined. It says “...26% with a score of 1.” There is no proper introduction of the ranges for a reader to comprehend the meaning of a score of 1. It also states that “...average efficiency score (TE-VRS) across all countries is 0.849 for VRS [variable returns to scale].”

**Response:** Thank you for this comment. We will address it.

10. The Discussion section needs revising. It does not directly connect to the findings of the study, despite the challenges of the results. The Discussion section further presents a couple of statistics, especially in the first and second paragraphs, without sources or a clear connection to the findings.

**Response:** Thank you for this comment. We will address it.

## Abbreviations

MNCH: maternal, newborn, and child health
